# Money Follows the Person and Informal Caregivers: Insights Into a New Stage of the Caregiving Career

**DOI:** 10.1093/geroni/igaa057.340

**Published:** 2020-12-16

**Authors:** Julie Robison, Noreen Shugrue, Chanee Fabius, Richard Fortinsky, Martha Porter, Kristin Baker

**Affiliations:** 1 University of Connecticut, Farmington, Connecticut, United States; 2 Johns Hopkins Bloomberg School of Public Health, Baltimore, Maryland, United States; 3 UConn Health, Farmington, Connecticut, United States

## Abstract

The Money Follows the Person (MFP) program transitions people to the community after extended institutional stays. This study examines effects of this transition on informal caregivers in this new caregiving career stage. Analyses explore whether and how MFP affects caregivers according to caregiver race/ethnicity, and care recipient age and disability type. Data come from surveys with 686 caregivers of persons in Connecticut’s MFP from November 2014-November 2018. Using Pearlin’s Caregiver Stress Process Model, bivariate and multivariate analyses examine predictors of multiple caregiver well-being indicators. Care recipients: older adults (50%), and younger persons with physical (35%), mental health (8%) or developmental (7%) disabilities. Caregivers: non-Hispanic White (62%), non-Hispanic Black (24%), and Hispanic (14%). Caregivers’ average assistance is 5 days/week, 6 hours/day, with 3 activities of daily living and 5 instrumental activities; 11% are paid for caregiving. Compared to other community-based samples, they report low mean levels of burden (4.7 of 16), anxiety (2.2 of 18) and depressive symptoms (31%), and high positive feelings about caregiving (9.5 of 12). A majority feel less stressed (60%) or no change in stress (20%) compared to before and during the institutional stay. Caregivers across the four care recipient groups don’t differ on most outcomes, although more caregivers of people with developmental disabilities (82% vs. 55-61%) report less stress once the person transitions. Black and Hispanic caregivers report more intensive caregiving, but White caregivers report more burden and subjective stress. Findings illustrate the benefits of programmatic support during a newly defined post-institutionalization caregiving career stage.

